# Influences of Infection Prevention and Control Practices for Transmissible Infections Among Operating Room Team Members: A Systematic Review

**DOI:** 10.1002/nop2.70956

**Published:** 2026-10-22

**Authors:** Kavitha Sriharan, Rona Pillay, Paul Glew, Gift Mutsonziwa

**Affiliations:** ^1^ School of Nursing and Midwifery Western Sydney University Sydney Australia

**Keywords:** barriers and facilitators, healthcare personnel, infection control, infection transmission, operating room, perioperative nursing, personal protective equipment, transmission‐based precautions

## Abstract

**Aim:**

To synthesise evidence on the influences of infection prevention and control practices for transmissible infections among operating room team members.

**Design:**

Systematic review.

**Data Sources:**

PubMed, CINAHL, APA PsycInfo, Web of Science and Scopus, supplemented by manual searches using Google Scholar and citation tracking.

**Review Methods:**

Databases were searched between March and April 2025, with an updated search undertaken in August 2026. Methodological quality was assessed using the Joanna Briggs Institute (JBI) critical appraisal tools and the Mixed Methods Appraisal Tool (MMAT). Data were synthesised using a convergent integrated approach.

**Results:**

Thirteen studies across quantitative, qualitative and mixed‐methods methodologies were included. Factors influencing infection prevention and control practices for transmissible infections among operating room team members included resource accessibility, organisational infrastructure and leadership support, alongside variation in knowledge and training. Precaution use differed across professional groups, whereas communication and teamwork were identified as important to coordinating infection prevention efforts. The practical demands of personal protective equipment also created challenges for communication and the delivery of surgical care. The four overarching themes were organisational support and resources; professional and team dynamics; knowledge and training; and the practical challenges in implementing precautions.

**Conclusion:**

Given the heterogeneity and predominantly observational nature of the evidence, the findings provide conceptual insight into factors influencing practice, although certainty regarding the effectiveness of specific strategies remains limited. Supporting infection prevention practices for transmissible infections in the operating room therefore requires attention to influences beyond individual knowledge, to include the wider team and organisational conditions in which precautions are implemented.

**Implications for Nursing Practice:**

Throughout a patient's operating room journey, perioperative nurses play a central role in coordinating patient care and infection prevention activities. They communicate infection risks, prepare the clinical environment, implement precautions, and coordinate care across professional groups. Support from nurse managers and clinical nurse educators through appropriate resources, continuing education, and interprofessional learning is important to sustain this role and promote consistent infection prevention practice for patient and staff safety.

**Impact:**

This review highlights the factors influencing infection prevention and control practices for transmissible infections among operating room team members. As transmissible infections continue to persist in healthcare settings, these findings may assist perioperative nurses and multidisciplinary teams to identify barriers and facilitators and inform locally relevant strategies that support consistent infection prevention practice and safer surgical care.

**Reporting Method:**

This systematic review was reported in accordance with the relevant EQUATOR guideline, the Preferred Reporting Items for Systematic Reviews and Meta‐Analyses (PRISMA) 2020 statement.

**No Patient or Public Contribution:**

No patient or public contribution was required as this systematic review synthesised data from previously published studies.

**PROSPERO Registration:**

CRD420251168178

## 1 Introduction

Infection prevention and control (IPC) practices are essential for maintaining patient and healthcare worker safety in clinical settings, particularly in the context of increasing antimicrobial resistance and emerging and re‐emerging infectious threats. In spite of the growing efforts to strengthen IPC practices, variation in adherence persists, particularly in high‐risk environments such as the operating room (OR). Given that IPC practices are central to patient safety and quality care, inconsistent adherence may increase the risk of infection transmission and compromise the safety of both patients and healthcare workers. The perioperative environment is complex, fast‐paced, and team‐based, requiring coordinated actions across multidisciplinary team members. Therefore, maintaining consistent application of IPC practices within this dynamic and time‐pressured environment remains challenging.

## 2 The Review

Effective infection prevention and control practices play a critical role in reducing healthcare‐associated infections (HAIs), and are widely recognised as a priority for patient safety and quality care (Australian Commission on Safety and Quality in Health Care [ACSQHC] 2017; World Health Organization 2016). Infection prevention and control practices are fundamental to reducing avoidable harm from pathogens and provide an evidence‐based approach to safeguarding both patients and healthcare workers (Magadze et al. 2022). The World Health Organisation's (WHO 2016) IPC initiatives emphasise strict adherence to standard and transmission‐based precautions (TBPs). Standard precautions provide the foundation for infection prevention and control practices. However, when patients are known or suspected to have infections or colonisation with pathogens that can be spread through contact, droplet, or airborne routes, additional transmission‐based precautions (TBPs) are required (World Health Organization 2016). For the purposes of this review, the term *transmissible infections* refers to infections or colonisation requiring additional precautions to prevent transmission between patients, healthcare workers and the healthcare environment.

Despite these efforts, healthcare‐associated infections (HAIs) remain a major global patient safety challenge. The World Health Organization (2022) estimates that 7% of patients in high‐income countries and 15% in low‐ and middle‐ income countries acquire at least one HAI during hospitalisation. One in 10 affected patients, on average, will die as a result of their infection (World Health Organization 2022). These infections continue to contribute substantially to preventable morbidity, prolonged hospitalisation, and rising healthcare costs worldwide. In Australia alone, HAIs are associated with an estimated 170,000 cases annually and over 7500 associated deaths (Lydeamore et al. 2022).

Patients undergoing surgery are among those at highest risk of healthcare‐associated infections (Sartelli et al. 2022). This vulnerability is attributed to the invasive nature of surgery, which breaches the body's natural defence barriers, together with multiple opportunities for pathogen transmission within the OR (Etherington et al. 2021; Facciolà et al. 2019). Potential reservoirs include healthcare workers' hands, anaesthesia equipment, monitors, operating tables, surgical instruments and other high‐touch surfaces (Boyce 2024; Schmutz et al. 2023). Multidrug‐resistant organisms (MDROs) may be transferred from colonised or infected patients to healthcare workers' hands and subsequently to equipment, the environment or other patients. Intraoperative transmission of methicillin‐resistant *Staphylococcus aureus* (MRSA), for example, has been associated with contamination of anaesthesia practitioners' hands and subsequent transmission between surgical cases (Loftus et al. 2018).

Achieving consistent IPC compliance in the OR is challenging due to the dynamic and sociocultural nature of perioperative care (Troughton et al. 2019). The operating room team includes surgeons, anaesthetists, instrument nurses (scrub nurses), circulating nurses (scout nurses or circulators), and operating assistants (orderlies) who work in close proximity to deliver coordinated patient care. Each team member performs distinct but interdependent roles, creating shared responsibility for maintaining infection prevention practices while balancing the immediate requirements of surgical and anaesthetic care. The combination of multidisciplinary teamwork, frequent movement, and high‐risk procedures increases the potential for infection transmission if precautions are not consistently implemented. For instance, airway management in general anaesthesia increases healthcare workers' exposure to respiratory secretions and potentially infectious aerosols, particularly during intubation and extubation (Alsofyani et al. 2020).

To support safe and consistent practice, IPC guidance draws on national and international standards. Key examples include the ACORN Standards for Perioperative Nursing in Australia (Australian College of Perioperative Nurses [ACORN] 2023), National Safety and Quality Health Service Standard 3 (Australian Commission on Safety and Quality in Health Care [ACSQHC] 2017), and the World Health Organisation guidelines on the core components of IPC programmes (World Health Organisation [WHO] 2016). These documents provide evidence‐based principles for reducing infection risk that can be applied within perioperative practice. However, despite the availability of such guidance, variations in practice are evident. Factors such as limited education, inconsistent enforcement of protocols, and variable awareness have been identified as potential contributors to the gap between recommended practices and their implementation within perioperative settings (Annis 2017; Kasatpibal et al. 2018). The importance of effective IPC practices has become increasingly evident in recent years with the rise of infection threats linked to emerging viral variants and the continuing challenge of MDROs (Igwe et al. 2024). The COVID‐19 pandemic also placed unprecedented demands on the healthcare workforce and resources, highlighting the need for reviewing response measures to disease outbreaks, particularly in high‐risk areas such as the OR (Ochie et al. 2022). These evolving infection threats reinforce the importance of consistently implementing appropriate precautions to protect both patients and healthcare workers from transmission within the OR.

Given the central role of nurses in coordinating perioperative care and maintaining IPC standards (ACORN 2023; ACSQHC 2017), understanding the factors that influence these practices is critical to informing nursing practice. However, existing evidence remains fragmented and is largely focused on specific professional groups, infection types or healthcare contexts. This makes it challenging to gain a comprehensive understanding of how IPC practices are enacted within the complex, multi‐disciplinary environment of the OR. In particular, there is a lack of synthesis examining the factors that influence IPC practices for transmissible infections among OR team members. Addressing this gap provides a base for improving clinical practice, optimising safe care delivery, and strengthening IPC strategies in high‐risk surgical environments.

## 3 Aim

To systematically search, synthesise and interpret findings from studies which investigated factors influencing the infection prevention and control practices of operating room team members in preventing and managing transmissible infections.

## 4 Methods

### 4.1 Study Design

This systematic review was conducted in accordance with the Joanna Briggs Institute (JBI) methodology for mixed‐methods systematic reviews (Lizarondo et al. 2024) and registered in the International Prospective Register of Systematic Reviews [(PROSPERO) ID: CRD420251168178]. To synthesise the quantitative and qualitative findings, a convergent integrated approach was used, following the JBI methodology for mixed methods systematic reviews (Stern et al. 2020). The Preferred Reporting Items for Systematic Reviews and Meta‐analyses (PRISMA) 2020 checklist (Page et al. 2021) was used to ensure transparency and guided the reporting process in the systematic conduct of this review. Ethical approval was not required as the study was conducted using data from existing published literature.

### 4.2 Search Methods

The electronic databases of PubMed, CINAHL, APA PsycInfo, Web of Science and Scopus were searched between March and April 2025. Following revision of the search strategy, updated searches were conducted across all databases in August 2026 to identify studies relating to IPC practices for transmissible infections among operating room team members. The search strategy was developed iteratively through testing and refinement of keywords and subject headings. Although all roles within the operating room were included, terms for orderlies yielded limited results and were not retained in the final search strategy.

The literature search included relevant MeSH/Subject Headings and keywords such as ‘surgeons’, ‘infection control’, ‘operating rooms’ and ‘infection prevention’ using Boolean operators (“AND”, “OR”) to combine and link results. The search strategy was adapted for each database using database‐specific subject terms, keywords and syntax. For example, the complete search strategy conducted in Scopus on 15 August 2026, which retrieved 977 records, was:

TITLE‐ABS‐KEY (“infection control” OR “infection prevention” OR “cross infection” OR “infection transmission” OR “transmission based precaution*” OR “transmission‐based precaution*” OR isolation OR precaution*) AND TITLE‐ABS‐KEY (“operating room*” OR “operating theatre*” OR “operating department*” OR “perioperative team*” OR “perioperative staff” OR “perioperative personnel”) AND TITLE‐ABS‐KEY (“operating room personnel” OR “operating room staff” OR “operating theatre staff” OR “surgical team*” OR “perioperative team*” OR “perioperative personnel” OR “perioperative staff” OR “operating room nurs*” OR “operating theatre nurs*” OR surgeon* OR anesthe* OR anaesthe*) AND (LIMIT‐TO (LANGUAGE, “English”)) AND PUBYEAR > 2009 AND PUBYEAR < 2027.

The complete search strategies for all databases, including the search dates and number of records retrieved, are provided in Appendix S1.

To ensure a comprehensive search, a manual search was also conducted using backward citation tracking alongside screening of highly cited studies using keyword matching on Google Scholar.

### 4.3 Inclusion and Exclusion Criteria

Inclusion and exclusion criteria were developed using the PICo framework (Population; Phenomenon of Interest; Context) (Stern et al. 2014). Clearly defined inclusion and exclusion criteria support transparency and reproducibility (Liberati et al. 2009). All four authors contributed to the development of these criteria to ensure consensus regarding study eligibility. To be included for synthesis, the studies had to examine the factors influencing IPC practices used to prevent or manage transmissible infections (phenomenon of interest) among operating room team members (context and population). Additional inclusion criteria required studies to be full‐text, original empirical studies (quantitative, qualitative, or mixed‐methods), published in English from January 2010 onwards. This timeframe was selected to capture IPC practices within the context of modern healthcare systems and evolving infection control standards. Literature reviews, unpublished studies and practice commentaries were excluded. Studies reporting abstract‐only findings were not considered, as they did not provide sufficient data for detailed evaluation. Other exclusions included studies that focused solely on clinical outcomes, epidemiological risk factors, surgical site infection, surgical asepsis, sterile technique, antibiotic prophylaxis, or other healthcare‐associated infections without a focus on transmissible infections or infectious exposure. Studies conducted outside the operating room setting, populations other than the operating room team members and studies published in languages other than English were also excluded. Detailed inclusion and exclusion criteria are presented in Table 1.

**TABLE 1:** Study inclusion and exclusion criteria according to PICo.

Category	Inclusion criteria	Exclusion criteria
Population	Operating room team members (e.g., nurses, surgeons, anaesthetists, orderlies)	Patients, administrative personnel or personnel not involved in intraoperative care
Phenomenon of interest	IPC practices undertaken to prevent or manage transmissible infections, including transmission‐based precautions (contact, droplet and airborne) and precautions for blood‐borne pathogens	Studies focused solely on surgical site infection, surgical asepsis, sterile technique, device‐associated infections (e.g., catheter‐associated infections), antibiotic prophylaxis, or other HAIs without a focus on transmissible infections or infectious exposure
Context	Operating room	Wards, ICUs, outpatient clinics, or general hospital settings

### 4.4 Search Outcomes

Database searching identified 2765 records, with five additional records identified through Google Scholar and citation searching. The database records were exported to EndNote 21, where 1003 duplicates were removed using a combination of automated tools and manual inspection. The remaining 1762 records were imported into Covidence (Veritas Health Innovation, n.d.). Titles and abstracts were screened by two reviewers against the predefined eligibility criteria, resulting in the exclusion of 1676 records.

Eighty‐six reports were sought for retrieval, of which one could not be retrieved. The remaining 85 reports, together with the five additional reports identified through Google Scholar and citation searching, underwent full‐text eligibility. Disagreements were resolved through discussion, with input from a third and fourth reviewer when required.

Following full‐text assessment, 77 reports were excluded. The main reasons were an infection prevention focus outside the scope of the review (*n* = 53), publication type (*n* = 20), an ineligible setting (*n* = 2), an ineligible population (*n* = 1), and a duplicate publication (*n* = 1). Thirteen studies met the inclusion criteria (Figure 1). Reports that appeared to meet the eligibility criteria but were subsequently excluded, together with the report that could not be retrieved, are presented with reasons in Appendix S2.

**FIGURE 1:**
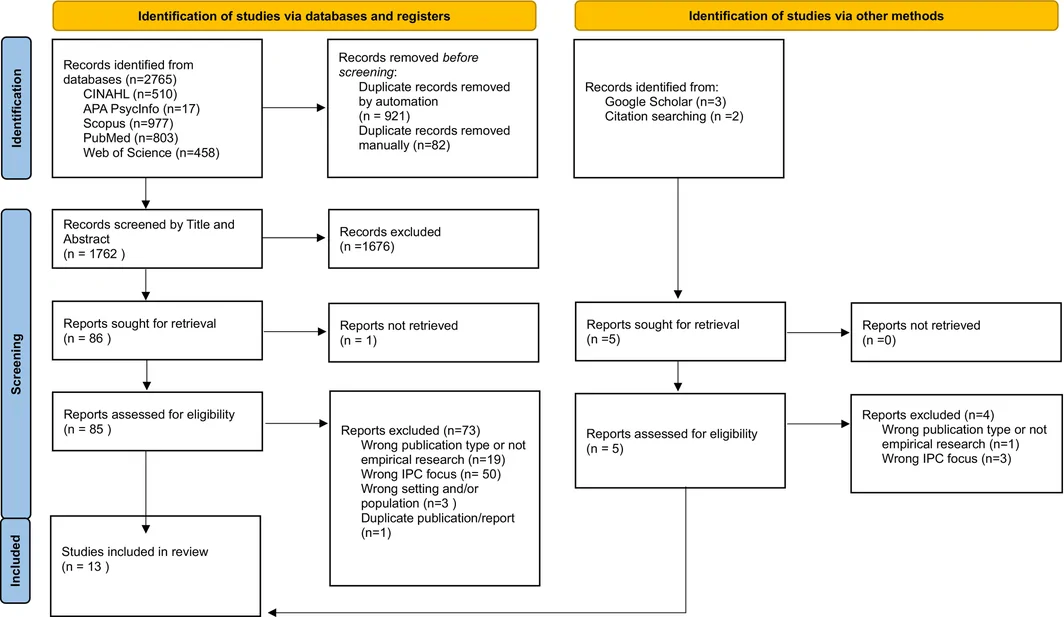
PRISMA 2020 flow diagram for new systematic reviews which included searches of databases, registers and other sources (Page et al. 2021).

### 4.5 Quality Appraisal

Critical appraisal was undertaken using design‐appropriate tools to assess methodological quality, including potential sources of bias relevant to each study design. The Joanna Briggs Institute critical appraisal tools were used for qualitative, analytical cross‐sectional, cohort, quasi‐experimental and randomised controlled studies (Barker et al. 2023, 2024, 2026; Lockwood et al. 2015; Moola et al. 2020). Mixed‐methods studies were assessed using the Mixed Methods Appraisal Tool (Hong et al. 2018).

Each JBI critical appraisal tool criterion was assessed as ‘Yes’, ‘No’, ‘Unclear’ or ‘Not applicable’. To summarise methodological quality, criteria rated ‘Yes’ were assigned one point, whereas ‘No’ and ‘Unclear’ responses received zero. Criteria considered ‘Not applicable’ were omitted from the denominator. Appraisal results were expressed as the percentage of applicable criteria rated ‘Yes’ to provide a descriptive summary and facilitate comparison across studies, as the number of criteria varied between JBI critical appraisal tools and study designs. The percentage was not used to determine overall risk of bias, classify study quality or exclude studies. Rather, to enable a comprehensive analysis of the literature, appraisal findings were considered during interpretation of the synthesis. The appraisal was conducted independently by two reviewers, with disagreements resolved through discussion and consensus. Findings from studies with considerable methodological limitations were interpreted cautiously and considered alongside the consistency of findings from studies with fewer identified limitations. As such, no studies were excluded based on the appraisal findings.

Overall, the 13 included studies provided useful but methodologically heterogeneous evidence, including cross‐sectional surveys, qualitative and mixed‐methods studies, observations, quality improvement studies, and a randomised controlled trial. A summary of the outcomes of the study appraisals is presented in Appendix S3. The differences in study designs and methods limited both direct comparison between studies and conclusions about the effects of infection prevention practices on infection outcomes.

The appraisal identified methodological limitations across several of the cross‐sectional studies. Common limitations included inadequate consideration of potential confounding factors (Belgacem et al. 2023; Eser and Aygün 2024; Lotfi et al. 2021; Prakash et al. 2020), with unclear reporting against several appraisal criteria also evident in some studies. In contrast, Ünal et al. (2025) met most applicable appraisal criteria. Among the intervention and environmental studies, Wall et al. (2022) and Munoz‐Price et al. (2012) met all applicable JBI criteria and had comparatively few identified methodological limitations. Chia et al. (2024) provided useful practice‐based evidence but were limited by the absence of a control group and uncertainty around participant comparability. However, Lacourciere et al. (2019) had more substantial methodological limitations and were therefore interpreted cautiously within the synthesis.

Among the qualitative studies, Elliott et al. (2021) met all JBI appraisal criteria, whereas limitations in Sürme et al. (2022) primarily concerned reporting of philosophical positioning and researcher reflexivity. The mixed‐methods study by Cutter and Jordan (2012) met all applicable MMAT criteria across its qualitative, quantitative, and mixed‐methods components, although the use of self‐reported data and response rate remained methodological limitations. The randomised controlled trial by Stutz et al. (2023) demonstrated true randomisation and appropriate outcome measurement and statistical analysis. However, limitations were identified in allocation concealment, blinding, and treatment group management. Overall, many studies were conducted within single centres and relied on observational, self‐reported, or non‐randomised designs, limiting generalisability of the findings. The evidence was better suited to identifying patterns and influences on IPC practices for transmissible infections than to establishing causal relationships between specific interventions and infection‐related outcomes.

### 4.6 Data Abstraction

Following quality appraisal, the data extraction process commenced. Data were systematically extracted from the chosen full‐text studies into a Microsoft Word data extraction form by two independent reviewers. Data extracted included: author, year of publication, country of research setting, aim, study design, methods, number/characteristics of participants, key findings (for quantitative studies), main themes (for qualitative studies), and study strengths/limitations. The extracted data were iteratively refined with discussion between the review team members to ensure consistent interpretation and categorisation across studies.

### 4.7 Synthesis

Data synthesis was undertaken in accordance with the JBI Mixed Methods Review Methodology Group (Lizarondo et al. 2024). Consistent with JBI guidance (Stern et al. 2020), a convergent integrated approach was adopted, as the research aim was best addressed through the integration of both quantitative and qualitative evidence. Once data extraction was completed, quantitative data (including the quantitative components from mixed‐methods studies) were transformed into textual descriptions or ‘qualitised’ (Sandelowski et al. 2006; Stern et al. 2020). This approach involved converting relevant percentages, differences between participant groups, and quantitative findings concerning barriers and facilitators into narrative statements (Sandelowski et al. 2006). These textual statements were then combined with the qualitative data and the qualitative components of mixed‐method studies. This was followed by a process of coding and categorising data based on their similarity in meaning, with each category consisting of two or more data elements. These categories were subsequently synthesised into themes and interpreted to produce the overall findings of the review in line with the research aim. During the data synthesis stage, regular team discussions facilitated a shared interpretation of concepts and themes, particularly where both qualitative and ‘qualitised’ data were integrated.

One reviewer conducted the initial transformation of quantitative findings into textual statements and coded the integrated qualitative and ‘qualitised’ findings. The transformed statements, categories and developing themes were subsequently reviewed by two members of the review team. Differences in interpretation or categorisation were discussed among the review team and resolved through consensus. Findings that differed or contradicted one another were retained and considered in relation to study design, professional group, setting and clinical context or resolved through majority agreement. An audit trail of the synthesis process, mapping transformed findings from contributing studies to the relevant categories and final themes, is provided in Appendix S4.

An assessment of the overall certainty or confidence in the synthesised evidence using GRADE or ConQual was not undertaken. Consistent with current JBI guidance, these approaches are currently not recommended for convergent integrated mixed‐methods systematic reviews because the transformation and integration of different forms of evidence present challenges for the grading process (Lizarondo et al. 2024). Furthermore, meta‐analysis was not undertaken because the quantitative studies did not provide sufficiently comparable estimates of common outcomes or intervention effects to support meaningful statistical pooling. The review instead sought to integrate findings at a conceptual level concerning factors influencing IPC practices for transmissible infections across different study designs and forms of evidence.

## 5 Results

### 5.1 Overview of the Included Studies

The main characteristics of the 13 included studies are summarised in Table 2. The studies were published between 2012 and 2025 and participants represented a range of intraoperative roles, including surgeons, anaesthetists, nurses and other operating room personnel, such as technologists. The studies were conducted across diverse geographical settings and sample sizes varied considerably according to study design. The evidence base included both multidisciplinary and profession‐focused studies. Six studies sampled multiple professional groups (Cutter and Jordan 2012; Elliott et al. 2021; Eser and Aygün 2024; Lotfi et al. 2021; Prakash et al. 2020; Stutz et al. 2023), whereas others focused primarily on nurses or anaesthesia personnel.

**TABLE 2:** Summary of included studies.

Author (s) (year) & country	Aim	Study design, methods & participants	Key findings (quantitative study)	Main themes (qualitative study)
Belgacem et al. (2023) Tunisia	To describe operating‐room nurses' knowledge, attitudes and practices regarding blood‐exposure accidents (BEAs) and to identify factors associated with these outcomes	*Quantitative study* Self‐administered questionnaire *Participants* *n* = 133 operating room nurses	Overall knowledge was moderate. Although 82.7% knew the meaning of blood exposure accidents (BEA), 17.3% did not. Most identified Hepatitis B and C as important blood‐transmitted infections (88.7%), whereas 69.2% identified HIV/AIDS Only 48.1% had received training on BEAs, whereas 90.2% wanted more knowledge on the topic	—
Chia et al. (2024) Singapore	To improve correct PPE handling among junior anaesthetists from approximately 20% to 80% in the operating theatres of Singapore General Hospital	*Quantitative study* Baseline self‐administered survey followed by intervention package *Participants* *n* = 36 junior anaesthetists	At baseline, 52.7% reported placing goggles on their forehead or on an unwiped surface immediately after intubation. 61.1% reported reusing disposable gowns for both intubation and extubation. 86.1% reported storing goggles incorrectly, such as in an uncleaned plastic bag or with a facemask. 64% felt that they lacked knowledge about PPE requirements for aerosol‐generating procedures, donning and doffing, disposal of single‐use PPE, and cleaning and storage of reusable PPE Root cause analysis identified the main reported causes of non‐compliance were concerns about waste generation, potential PPE shortages, practices interfering with the care of unstable patients, inconvenience, and discomfort Following interventions, correct goggle handling after intubation increased from 33.3% in Week 1 to 78.5% in Week 19. Correct goggle storage and face‐shield disposal increased from 13.6% to 78.6%	—
Cutter and Jordan (2012) Wales	To identify the frequency of self‐reported adverse exposure to blood and body fluids among surgeons and scrub nurses during surgical procedures; contributory factors to such injuries; the extent of compliance with standard precautions; and factors influencing compliance with precautions	*Mixed methods study* Postal surveys, semi‐structured and telephone interviews *Participants* *n* = 315 (surgeons and scrub nurses) *n* = 6 (senior infection control nurses)	31 of 315 respondents (9.8%) reported consistent adherence to all available standard precautions Precaution use was frequently adjusted according to perceived patient risk: 58.1% agreed that staff took fewer precautions with patients considered low risk, whereas 66.8% reported using double gloves only for patients with known or suspected blood‐borne infection Scrub nurses reported higher use of eye protection for all patients than surgeons: 62.7% (84/134) vs. 32.4% (57/176). Surgeons were significantly less likely to use eye protection than scrub nurses (OR 0.28, 95% CI: 0.11–0.71)	Differences in risk perception Surgeons and scrub nurses had differing perceptions of risk of acquiring blood‐borne viral (BBV) infections, with surgeons acknowledging the belief of sharps' injuries as an expected part of their job Training and awareness gaps Education and training sessions were poorly attended, particularly by surgeons, who appeared to lack awareness of the risk of BBV infection and expressed scepticism about the effectiveness of these sessions in improving compliance
Elliott et al. (2021) United Kingdom (UK) Italy Spain United States of America China New Zealand	To explore the experiences and innovations developed by international surgical teams during the early stages of the COVID‐19 pandemic to attempt to mitigate risks	*Qualitative study* Semi‐structured interviews *Participants (n = 43)* surgeons, anaesthetists, and nurses	—	Effective IPC was underpinned by strong team communication and collaboration. Regular discussions, use of digital platforms, and shared problem‐solving helped manage risk, reflecting the critical role of organisational and team dynamics in IPC adherence Challenges with PPE such as discomfort, impaired communication and procedural difficulties influenced IPC compliance compelling staff to prioritise operability over full protection
Eser & Aygün. (2024) Türkiye	To evaluate the adverse effects of multilayered COVID‐19 PPE used in operating rooms across six areas: respiratory, visual, hearing and communication, thermal stress, movement and occupational skills, and neurocognitive and psychological effects	*Quantitative study* Face‐to‐face interviews and self‐reported questionnaires *Participants* *n* = 89 (doctors) *n* = 67 (operating room nurses) *n* = 47 (anaesthesia technicians)	91.6% reported reduced respiratory quality. 94.1% reported reduced visual quality. Among those reporting visual problems, 89.2% experienced visor fogging, 54.7% reported light reflection and 29.6% reported narrowing of the visual field 61.6% reported reduced hearing quality. 93.6% reported problems communicating within the surgical team 96.1% reported increased sweating. 95.1% reported increased warmth. 95.1% reported increased fatigue. 85.2% reported reduced psychological tolerance, described as reduced patience or endurance 85.2% had not received training on breathing with an N95 mask, 91.6% had not received training on head movements to improve vision and prevent balance problems, and 90.6% had not received training to prevent PPE‐related dehydration 9.9% reported having routine access to rest in an oxygen‐rich or open‐air area after surgery, and 13.3% reported having the opportunity to shower after procedures involving COVID‐19 PPE	—
Lacourciere et al. (2019) United States of America	To develop and implement an evidence‐based Operating Room Contact Precautions Decision Algorithm (CPDA)	*Quantitative study* Post‐implementation survey *Participants* *n* = 27 perioperative nurses	66.6% agreed or strongly agreed that they knew where to find the CPDA. 77.8% agreed or strongly agreed that the CPDA was easy to follow and this was associated with the perception that CPDA improved efficiency (*χ* ^2^ = 6.259 and *p* = 0.012) 70.4% agreed or strongly agreed that they experienced less pushback from surgeons when requesting terminal cleaning 100% agreed or strongly agreed that they were supported by the charge nurse and operating room leaders when terminal cleaning with or without UV‐C was required	—
Lotfi et al. (2021) Iran	To determine the level of compliance with preventive standards against COVID‐19 transmission in operating rooms	*Quantitative study* Self‐report questionnaire *Participants (n = 183)* *n* = 121 operating room nurses *n* = 24 surgeons *n* = 38 surgical residents	The main sources of knowledge about COVID‐19 preventive measures were: colleagues (38.25%), articles and guidelines (37.70%), training workshops (13.66%) Overall PPE adherence was rated as favourable (mean 61.8 ± 8.1), whereas adherence to broader operating room COVID‐19 protective measures was moderate (mean 55.3 ± 10.5) Hand hygiene after patient/environmental contact (3.8 ± 0.39) and appropriate glove use (3.6 ± 0.61) were among the most frequently reported practices Mean PPE adherence scores differed by professional group, with lower mean scores among surgical residents (58.05) than operating room nurses (62.67) and attending surgeons (63.83) (one‐way ANOVA, *p* = 0.004; Tukey post hoc comparisons: residents vs. nurses, *p* = 0.006; residents vs. attending surgeons, *p* = 0.01)	—
Munoz‐Price et al. (2012) United States	To determine the change in both the thoroughness of environmental cleaning and the proportion of environmental surfaces within the OR from which pathogenic organisms were recovered	*Quantitative, quasi‐experimental, prospective study* UV markers, environmental cultures, verbal and graphic educational programs following feedback *Participants* Environmental services personnel, and OR technicians involved in the cleaning and monitoring of 194 operating rooms and 2820 objects	Structured education and performance feedback programme led to a significant improvement in operating room cleaning, increasing cleaning thoroughness from 47% (95% CI: 0.42–0.53) to 82% (95% CI: 0.77–0.85) (*p* < 0.0001) The percentage of surfaces from which gram‐negative bacilli were recovered decreased from 10.7% at baseline to 2.3% at follow‐up (*p* = 0.015)but there was no overall pathogen reduction during the study period Anaesthesia equipment cleaning improved from 25% to 77% following the reassignment of cleaning duties to the anaesthesia team	—
Prakash et al. (2020) India	To audit the compliance and perception of personal protective equipment (PPE) among health care workers involved in the surgery of COVID‐19 negative cancer patients during the pandemic in a single, high‐volume, tertiary cancer centre	*Quantitative study* Observational audit using an institution‐tailored checklist followed by surveys *Participants: Observations/involvements across 183 surgeries* 567 surgical team members 413 anaesthesia team members 291 scrub nurses 164 OR technicians	Healthcare workers in the OR demonstrated a high overall PPE compliance rate of 96.3% The most frequent lapses in adherence were with eye protection, particularly among surgeons (45/567 of respondents, *p* < 0.001), mostly due to discomfort, and fogging of visors	—
Sürme et al. (2022) Türkiye	To explore the experiences of operating‐room nurses caring for patients who were COVID‐19 positive or suspected of having COVID‐19 during the surgical process	*Qualitative study* Face‐to‐face, semi‐structured interviews *Participants* *n* = 22 operating room nurses	—	Difficulties experienced in the operating room: Nurses reported fear of contracting COVID‐19 and transmitting it to their families. Also described shortages and discomfort associated with PPE. Emergency operations were particularly difficult because there was often insufficient time to test patients for COVID‐19 or to put on protective equipment safely The effect of the pandemic on operating‐room organisation: Elective operations were postponed, operation times were shortened and longer intervals were introduced between operations for disinfection. Separate patient‐transfer routes, elevators and operating rooms were also created for COVID‐19‐positive or suspected patients Effect of transmission risk on surgical‐team communication: Social distancing, staff rotation and PPE reduced communication and increased feelings of isolation and anxiety. Difficulty hearing and communicating while wearing protective equipment also contributed to nurses' fear of making medical errors
Stutz et al. (2023) Switzerland	To compare the effectiveness of simulation‐based training versus video‐based training on hygiene knowledge, psychological safety, and perceptions of isolation measures among healthcare workers in the operating room	*Quantitative—prospective, multi‐centre randomised controlled trial* Participants randomly allocated to: simulation‐based training OR video‐based training Pre‐ and post‐intervention questionnaires *Participants* *n* = 54 in simulation group *n* = 31 in video group Physicians (registrars and consultants) and anaesthetic nurses	Neither simulation training nor video‐based training led to a significant improvement in hygiene knowledge (*F* = 4.049, *p* = 0.157, partial *η* ^2^ = 0.024) or perceived psychological safety overall (*F* = 0.235, *p* = 0.629, partial *η* ^2^ = 0.003) Participants rated the simulation‐based training more positively than the video‐based training (*M* = 5.15 ± 0.65 vs. 3.67 ± 1.04, *p* < 0.001) Participants in the simulation group also reported a higher willingness to speak up	
Ünal et al. (2025) Türkiye	To determine the awareness and knowledge levels of operating‐room staff regarding the precautions required during the COVID‐19 pandemic	*Quantitative study* Introductory Information Form and COVID‐19 Operating Room Precaution Form based on WHO guidance *Participants* *n* = 102 operating room staff members	96.1% supported preparing information materials about operating‐room hygiene and the correct use of personal protective equipment, whereas 93.1% supported regular training Regarding operating‐room organisation, 97.1% supported using a separate operating room for patients with a confirmed or suspected COVID‐19 diagnosis. A further 87.3% supported assigning additional personnel to provide materials during surgery 95.1% stated that appropriate PPE should be used in the OR. 98% supported minimising the number of staff in the operating room and limiting movement in and out of the room With regard to cleaning and waste management, 57.8% selected the recommended procedure for disinfecting medical‐device surfaces, and 52.9% selected the recommended sodium‐hypochlorite cleaning procedure	—
Wall et al. (2022) United States	To assess the effectiveness and feasibility of a post‐implementation evidence‐based intraoperative infection control program targeting improved basic measures	*Quantitative, prospective, case‐cohort study* Environmental sampling, clinical observations and patient follow‐up *Participants* *n* = 801 surgical patients *n* = 23 ORs (anaesthesia providers implemented the interventions and were monitored for compliance)	The intervention reduced environmental contamination, *Staphylococcus aureus* transmission (from 24% to 9%, IRR 0.40, 95% CI: 0.28–0.56) and SSIs (from 8% to 3%; adjusted IRR 0.45, 95% CI: 0.25–0.82) and maintained effectiveness across heterogenous OR settings The intervention was associated with improved hand‐hygiene and environmental‐cleaning compliance, indicated by reduced contamination of provider hands and anaesthesia‐environment reservoirs. These improvements were sustained during six months of post‐COVID‐19 follow‐up	—

*Note:* ‘—’ indicates that this section was not included in the study.

The evidence was predominantly quantitative (*n* = 10), with two qualitative studies and one mixed‐methods study. Eight studies were conducted in the context of the COVID‐19 pandemic (Chia et al. 2024; Elliott et al. 2021; Eser and Aygün 2024; Lotfi et al. 2021; Prakash et al. 2020; Stutz et al. 2023; Sürme et al. 2022; Ünal et al. 2025). Synthesis of the findings generated four overarching themes: organisational support and resources; professional and team dynamics; knowledge and training; and practical challenges in implementing precautions.

### 5.2 Theme 1: Organisational Support and Resources

The practical conditions created by healthcare organisations influenced how readily IPC practices could be implemented in the OR. In a single‐centre Turkish study of OR team members during the COVID‐19 pandemic, Eser and Aygün (2024) reported that inadequate organisational facilities limited staff recovery from the effects of prolonged PPE use. Access to open‐air or oxygen‐rich rest areas and opportunities to shower after surgery were limited. Consequently, OR team members experienced difficulty coping with PPE‐related problems, which contributed to avoidance of PPE use. In contrast, Sürme et al. (2022) reported that nurses perceived adequate staffing, reasonable working hours and longer intervals between procedures as conditions required to effectively implement IPC protocols during the COVID‐19 pandemic. Designated operating rooms and separate transfer pathways for COVID‐19 patients also provided greater workflow clarity for OR nurses and increased their sense of safety.

The availability of infrastructure and PPE also differed across OR settings. In a multi‐centre study of OR team members across teaching hospitals in Iran, Lotfi et al. (2021) reported differences in the implementation of COVID‐19 precautions related to OR infrastructure and organisation. Signage and restrictions on OR traffic and overcrowding were among the more commonly implemented measures, whereas recommended ventilation and negative‐pressure requirements were less consistently implemented. Among junior anaesthetists at a large tertiary hospital in Singapore, Chia et al. (2024) identified concerns about potential PPE shortages as an important perceived reason for non‐compliance during the COVID‐19 pandemic. In the context of supply chain disruptions and misinformation, these concerns contributed to inappropriate PPE reuse and storage. However, following an intervention that modified PPE storage and improved access to disinfectant wipes, PPE handling practices improved.

Organisational oversight and support also contributed to how IPC practices were maintained and reinforced. In a large single‐centre study in the United States, Munoz‐Price et al. (2012) used environmental monitoring to assess the thoroughness of OR cleaning, with staff receiving verbal and visual education and performance feedback. Cleaning subsequently improved, with the greatest improvement observed for anaesthesia equipment following reassignment of cleaning responsibility to the anaesthesia team. However, improvements in cleaning were not accompanied by an overall reduction in environmental pathogen contamination. Wall et al. (2022) similarly incorporated surveillance monitoring and feedback into a multifaceted intraoperative IPC programme. Bacterial transmission was monitored and monthly feedback was used to guide ongoing optimisation of the programme, which was associated with reductions in *Staphylococcus aureus* ( *S. aureus* ) transmission. Chia et al. (2024) used repeated education, direct observation, feedback and reminders to reinforce PPE handling and storage practices among junior anaesthetists during the COVID‐19 pandemic, alongside broader departmental communication and support. Lacourciere et al. (2019) also identified nursing leadership as a source of organisational support, with perioperative nurses reporting consistent support from nursing leaders when terminal cleaning was required for patients on contact precautions.

### 5.3 Theme 2: Professional and Team Dynamics

Infection prevention and control practices varied across professional groups, with role‐specific differences reported in measures intended to reduce infection transmission. Three studies (Cutter and Jordan 2012; Prakash et al. 2020; Lotfi et al. 2021) identified variation in hand hygiene and personal protective equipment use. In a UK study comparing surgeons and scrub nurses, Cutter and Jordan (2012) found that surgeons were less likely than scrub nurses to use eye protection. The two professional groups also differed in their perceptions of infection risk and application of precautions for blood‐borne infections. At a large tertiary‐care cancer centre during the COVID‐19 pandemic, Prakash et al. (2020) observed that scrub nurses demonstrated the highest adherence to prescribed PPE, whereas face‐shield non‐compliance occurred predominantly among surgeons. Similarly, Lotfi et al. (2021) reported differences in PPE adherence by professional group, with attending surgeons and nurses demonstrating higher adherence than surgical residents.

Beyond these professional differences, effective management of transmissible infection risks also depended on team communication and collaboration. In an international, multi‐centre study by Elliott et al. (2021), surgical teams described communication and teamwork as important when adapting practice to reduce COVID‐19 transmission, particularly as recommendations changed rapidly. Teams used regular discussions, defined staff roles and patient pathways, buddy systems and simulation to coordinate the implementation of precautions. Prakash et al. (2020) similarly incorporated multidisciplinary briefings and debriefings into COVID‐19 surgical practice as a structured mechanism for interprofessional communication. This allowed team members to identify PPE errors and reinforce appropriate precautions.

Where practices were unclear or inconsistent, interprofessional collaboration and colleague influence became particularly important. Lacourciere et al. (2019) reported friction between perioperative nurses, surgeons and environmental services personnel regarding post‐procedure cleaning protocols for patients with multidrug‐resistant organisms (MROs) or *Clostridium difficile* . Nurses experienced pressure to maintain efficient OR turnover, whereas surgeons raised concerns about delays associated with enhanced cleaning. In response, an interprofessional team, including perioperative nurses, infection preventionists and environmental services personnel, with input from surgeons, developed a decision algorithm to standardise cleaning requirements. Following implementation, nurses reported experiencing less surgeon pushback when terminal cleaning was required, although perceptions of surgeon support were not unanimous. Chia et al. (2024) similarly demonstrated the influence of colleagues on IPC practice through a behaviour change intervention. The authors reported improved PPE compliance among junior anaesthetists when senior anaesthetists observed their PPE practices and provided feedback. When non‐ideal PPE practices were also observed among senior anaesthetists, education was extended to senior anaesthetists and OR nurses to encourage modelling of appropriate practices.

### 5.4 Theme 3: Knowledge and Training

Knowledge and preparedness influenced how OR team members applied precautions for transmissible infections. In a single‐centre study of operating room staff in Turkey, Ünal et al. (2025) identified high overall awareness of COVID‐19 infection control. However, gaps remained in knowledge of specific practices, particularly environmental cleaning. In spite of these gaps, participants strongly supported the use of information materials for correct PPE use, as well as the need for regular training. Lotfi et al. (2021) further found that OR team members primarily obtained knowledge of COVID‐19 protective measures from colleagues and written articles or guidelines, whereas formal educational activities such as workshops and webinars were less commonly reported as sources of knowledge. Similarly, Eser and Aygün (2024) identified a gap in formal education, finding that despite prolonged use of COVID‐19 PPE, operating room staff had not received additional training to manage associated difficulties with breathing, vision and balance. Knowledge and training needs were also evident in relation to occupational blood‐borne infection. Among OR nurses across two hospitals in Tunisia, Belgacem et al. (2023) found that gender and professional experience were associated with aspects of nurses' knowledge of blood exposure accidents. Female nurses demonstrated greater awareness of blood‐borne infection risks, whereas professional experience was associated with knowledge of specific risks related to blood exposure. Less than half of the nurses had received training on blood exposure accidents although the majority of them expressed a strong desire for further knowledge on the topic.

How education was delivered was also important, although this did not consistently translate into improved knowledge or practice. In a multi‐centre study in Switzerland, Stutz et al. (2023) compared simulation‐based and video‐based training for contact, droplet and airborne isolation measures in the OR. Neither training approach significantly improved participants' knowledge. However, simulation‐based training was perceived more positively and participants reported greater willingness to speak up when a senior colleague did not follow the appropriate IPC practice in the simulated scenario. Chia et al. (2024) used a different approach, incorporating repeated and multimodal education into an intervention addressing COVID‐19 PPE practices among junior anaesthetists. Educational messages and posters were supplemented by direct observation and face‐to‐face feedback. Education was also extended to senior anaesthetists and OR nurses when non‐ideal practices were identified more broadly. PPE compliance improved during the 19‐week intervention.

### 5.5 Theme 4: Practical Challenges in Implementing Precautions

The practical demands of using PPE created challenges for implementing IPC practices during the COVID‐19 pandemic. Eser and Aygün (2024) reported difficulties with breathing, vision and balance among OR team members who were required to wear PPE for prolonged periods. A majority of surgical team members also experienced reduced quality of communication while wearing COVID‐19 PPE in addition to difficulty hearing other team members. Prakash et al. (2020) similarly identified discomfort and fogging of face shields as reasons for non‐compliance with eye protection. Sürme et al. (2022) further found that PPE affected communication within surgical teams. Difficulty hearing and communicating while wearing protective equipment also contributed to nurses' concerns about making medical errors.

These practical difficulties were found to compete with the demands of delivering surgical care. Elliott et al. (2021) found that OR team members experienced difficulties maintaining recommended PPE practices while performing procedures. In addition to hindering communication, participants reported that PPE occasionally impaired both dexterity and the use of equipment during procedures, creating challenges in maintaining protection while operating effectively. Chia et al. (2024) similarly identified practical barriers to COVID‐19 PPE use among junior anaesthetists. Inconvenience and discomfort were perceived as reasons for non‐compliance, alongside concerns that some PPE practices could interfere with the care of unstable patients.

## 6 Discussion

This systematic review explored the factors influencing the IPC practices for transmissible infections among operating room team members. The findings highlighted how IPC practices are shaped by a combination of organisational, professional, educational and practical influences. Together, these factors reflect the multifaceted nature of perioperative practice, where adherence to IPC is not solely determined by knowledge, but by the interaction between individuals, teams and the systems within which they work.

*Organisational support and resources* emerged as important influences on the implementation of IPC practices for transmissible infections within the operating room. The reviewed studies indicated that adequate infrastructure, access to PPE, staffing and workflow arrangements, organisational oversight and leadership support could enable or constrain the implementation of precautions. This places emphasis on the conditions in which OR team members are expected to practise, rather than viewing IPC adherence solely as an individual responsibility. Similar findings have been reported by Alidina et al. (2021), who found that higher‐performing surgical facilities in Tanzania were characterised by stronger teamwork and communication, collective learning and engaged leadership. These organisational conditions supported the translation of the Safe Surgery 2020 intervention into sustained changes in practice and organisational processes. Likewise, qualitative research among OR nurses by Markström et al. (2026) identified supportive leadership, organisational structures, communication systems, staffing, and feedback as conditions that facilitated infection prevention practice. It also reported that time pressure, poor information transfer, unstable routines, and insufficient feedback undermined practice. These findings are also consistent with national and international expectations for perioperative nursing practice. Nationally, the ACORN Standards emphasise infection prevention, effective team communication and safe, evidence‐informed perioperative care (ACORN, 2026). Internationally, the International Federation of Perioperative Nurses (IFPN 2019) promotes safe surgery through evidence‐based practice, education, collaboration and the responsibility to safeguard patients. For perioperative nurses, meeting these expectations in practice depends on supportive organisational conditions, including effective nursing leadership.

The findings also highlight a potential gap between established IPC requirements and the organisational capacity to implement them in practice. Sax et al. (2020), in a Swiss national survey, found that although hospitals generally had established IPC policies, infection prevention staffing was frequently below international recommendations and leadership support was perceived as modest. More recently, Coffey et al. (2026) identified limitations in frontline training, isolation capacity, adherence to contact precautions and management of environmental reservoirs as barriers to MDRO prevention and control. This reflects findings in the present review, where infrastructure and resource constraints were reported across geographically diverse settings. However, the nature of these constraints differed between healthcare systems, suggesting that organisational barriers may not operate uniformly across settings. Resource shortages reported during the COVID‐19 period should also be interpreted within the exceptional supply and workforce pressures experienced at that time.

Importantly, intervention studies combining resource accessibility, education, monitoring and feedback reported improvements in PPE handling, environmental cleaning and intraoperative transmission outcomes (Chia et al. 2024; Munoz‐Price et al. 2012; Wall et al. 2022), although their multicomponent designs make it difficult to determine which individual strategies contributed to these improvements. This provides some support for organisational conditions as potentially modifiable influences on IPC practice, although there is less certainty about the effectiveness of individual organisational strategies.

*Professional and team dynamics* emerged as important influences on IPC practice within the OR. Across the reviewed studies, differences in precaution use were observed between professional groups in the implementation of IPC practices for preventing transmissible infections, particularly in relation to PPE use and hand hygiene (Cutter and Jordan 2012; Lotfi et al. 2021; Prakash et al. 2020). These findings suggest that IPC recommendations may not be interpreted or implemented uniformly across the multidisciplinary OR team. Similar professional variation has been identified by Deslandes et al. (2025), who reported differences between surgeons and anaesthetists in their knowledge, professional orientations, and approaches to infection prevention. Surgeons were described as more focused on immediate tasks and individual practice, whereas anaesthetists more often adopted collective and systems‐oriented approaches. Schutte et al. (2025) likewise identified variation in physicians' IPC decision‐making, with some relying on habitual judgements and others weighing infection risk against the perceived costs and benefits of precautions. Their decisions were influenced by personal beliefs, social norms, and features of the clinical environment.

Professional differences, however, occur within an interdependent team environment. During the COVID‐19 pandemic, Elliott et al. (2021) and Prakash et al. (2020) reported how multidisciplinary discussion, defined responsibilities and structured briefings supported the implementation of precautions. Similarly, Surendran et al. (2024) demonstrated how communication patterns and professional roles shaped coordination within surgical teams, with scrub nurses acting as important mediators across professional boundaries. Conversely, breakdowns in planning and information sharing between surgeons and other team members were associated with increased door openings and disruption of environmental controls. This is consistent with Markström et al. (2026), who found that nurses' ability to raise infection‐prevention concerns was supported by trust, respect, effective communication and recognition of their professional expertise. In contrast, unclear responsibilities, competing priorities and poor communication could undermine practice. These findings reinforce the interdependent nature of perioperative infection prevention efforts, where the ability of one team member to act may depend on how effectively concerns, responsibilities and expectations are communicated and coordinated across the team. For nursing practice, this highlights the importance of communicating concerns and working across professional boundaries while maintaining shared team responsibility. However, variation in the practices and roles examined, together with the predominance of studies from the COVID‐19 period, means that specific professional differences should be interpreted cautiously.

*Knowledge and training* emerged as important influences on how operating room team members implemented IPC practices for transmissible infections. Although some studies reported high general awareness of infection prevention, gaps remained in knowledge of specific practices and engagement with formal training (Belgacem et al. 2023; Eser and Aygün 2024; Lotfi et al. 2021; Ünal et al. 2025). This suggests that general awareness of infection risk does not necessarily translate into knowledge of how to apply specific precautions. Similar findings were reported by Cawich et al. (2013), where most staff considered themselves knowledgeable about infection prevention despite knowledge being commonly acquired through informal sources and adherence to infection control policies remaining low. This distinction may be particularly important for transmissible infections, where staff may need to apply specific precautions according to the mode of transmission and clinical situation.

Importantly, providing education did not necessarily translate into improved knowledge or practice. Within the reviewed studies, Stutz et al. (2023) found no significant improvement in knowledge following either simulation‐ or video‐based training for contact, droplet and airborne precautions, although simulation was perceived more positively. In contrast, Chia et al. (2024) reported improved PPE compliance following a multimodal intervention combining education with observation, feedback and broader team involvement. This variability suggests that the translation of IPC knowledge into practice may depend on influences beyond education alone. Curtis et al. (2022), in a scoping review of behaviour change approaches to IPC, identified knowledge as one of several behavioural determinants, alongside influences such as environmental context, beliefs about consequences and professional role. This is also reflected in intervention studies. Khan et al. (2021) identified improvements in knowledge and self‐reported behaviour following interprofessional training, but reported barriers to applying this knowledge in practice, including staff turnover, limited supplies and established practices. Similarly, van Dijk et al. (2023) found that management team training was not associated with improved compliance with a surgical site infection bundle, where limited clinician involvement and few subsequent improvement activities constrained implementation. These studies suggest that education may strengthen knowledge, but its translation into IPC practice is dependent on the behavioural, social or organisational influences that enable new knowledge to be applied (Chia et al. 2024; Curtis et al. 2022; Khan et al. 2021; van Dijk et al. 2023). For nursing education, this means that education needs to be practical, team‐based and supported by opportunities to apply learning in practice. However, the variable outcomes and methodological limitations among the intervention studies in this review provide less certainty about which educational approaches are effective in achieving sustained practice change.

*Practical challenges associated with implementing precautions* were evident across the reviewed studies, particularly in relation to PPE during the COVID‐19 pandemic. These difficulties were notably apparent even where overall precaution use was high. Prakash et al. (2020), for example, reported high overall PPE compliance, whereas discomfort, poor visibility and fogging contributed to lower adherence to eye protection. Similar practical difficulties involving communication, visibility, dexterity and equipment use were identified across other studies (Eser and Aygün 2024; Elliott et al. 2021; Sürme et al. 2022). This suggests that difficulties implementing precautions may reflect the practical demands of integrating protective measures into patient care, rather than knowledge or willingness to comply alone.

This pattern has also been identified in wider research. Yánez Benítez et al. (2020), in an international survey that examined surgeons performing emergency surgery, found that respirators muffled speech, whereas face shields interfered with voice projection. The surgeons reported that goggles and shields could fog or create glare, reducing visibility, and that these effects could interfere with communication, situational awareness, psychomotor performance, and decision‐making. From a human factors perspective, Hignett et al. (2021) conceptualised PPE as changing the ‘task interface’, meaning that the equipment alters how healthcare workers interact with patients, instruments, colleagues, and technology. Chasco et al. (2023) further demonstrated that correct PPE use may be affected by equipment familiarity and design. When equipment was unfamiliar or non‐intuitive, participants reported that the design itself and lack of training made correct doffing more difficult, and some deviated from best practice. Collectively, the evidence suggests that effective precaution use may depend not only on whether healthcare workers know what is required, but also on whether protective equipment can be readily and safely incorporated into the clinical tasks they are expected to perform. For nursing practice, this highlights the need to consider how PPE design and usability affect communication and the delivery of surgical care.

Although practical PPE‐related challenges were identified across several studies, their wider applicability remains less certain because much of the evidence was generated during the COVID‐19 pandemic and involved enhanced PPE use. These findings should therefore be interpreted cautiously when considering routine precautions for other transmissible infections.

### 6.1 Implications

Although the heterogeneity of study designs, settings and outcomes limits firm conclusions about the effectiveness of specific strategies, the synthesis provides conceptual insight into the factors that may influence IPC practices for transmissible infections in the operating room. These findings highlight organisational, professional, educational and practical influences that should be considered when developing context‐appropriate strategies to support IPC practice.

#### 6.1.1 Implications for Operating Room Team Practice

At an organisational level, IPC policies and implementation strategies should account for the conditions in which precautions for transmissible infections are applied in the operating room. Engagement with perioperative nurses and other frontline operating room team members may help identify local barriers relating to communication, resources, workflow and team practices. This may support the translation of IPC recommendations into workable operating room practice.

The findings also have implications for interprofessional education. Although knowledge and training were important influences on IPC practices for transmissible infections, education alone did not consistently translate into practice. Education should therefore include practical, context‐specific learning that is relevant to the operating room environment. Interprofessional education may support a shared understanding of transmission risks, the required precautions, and individual and collective responsibilities for their implementation. Learning should be reinforced through feedback and opportunities to apply it in clinical practice.

#### 6.1.2 Implications for Nursing Practice

Perioperative nurses can support the early identification and communication of infection risks by reviewing alerts and required precautions during operating list review and perioperative handover. The planned precautions can be reconfirmed with the multidisciplinary team during the team briefing or surgical time‐out. Coordinating these precautions may also involve informing the relevant operating room and recovery team members, ensuring that appropriate PPE and other resources are available, and preparing the clinical environment before the patient arrives. During and following the procedure, nurses can reinforce the agreed precautions, identify difficulties involving equipment or workflow, and escalate unsafe or inconsistent practices to nurse leaders and infection prevention personnel. Their contribution may also extend to leading IPC initiatives, acting as IPC champions, participating in audit and feedback, supporting staff education and advocating for safe practice.

Nurse managers can support these activities by ensuring appropriate staffing and resources, establishing clear communication and escalation processes, and encouraging multidisciplinary participation in infection prevention. Perioperative nurse educators can provide practical, case‐based education through simulation, familiarisation with protective equipment and observation of precaution use. Brief multidisciplinary debriefing after cases involving transmission‐based precautions may provide an opportunity to identify problems, share feedback and agree on improvements for future cases. These activities are consistent with national and international expectations for evidence‐based, collaborative and safe perioperative practice (ACORN 2026; IFPN 2019). Although perioperative nurses may initiate and coordinate these activities, their successful implementation remains a shared responsibility across the operating room team.

### 6.2 Strengths and Limitations

The strength of this review is evident in its adherence to rigorous methodology and reporting standards set by PRISMA and the Joanna Briggs Institute for mixed‐method reviews. The inclusion and integration of quantitative, qualitative, and mixed‐methods evidence enabled factors influencing IPC practice for transmissible infections to be examined across different professional groups and geographical contexts. Methodological quality was assessed using design‐appropriate critical appraisal tools, and the contribution of individual studies to the synthesis was considered alongside their methodological limitations.

Although this review has several strengths, a few limitations were also identified. The exclusion of non‐English language and unpublished research introduces the potential for language and publication bias. In addition, a preliminary search using broader terms identified many nursing studies from other clinical areas that were not related to the operating room. The search was therefore narrowed to align with the PICo framework and the focus of the review. However, this may have limited the number of relevant studies identified. Furthermore, the evidence base was methodologically heterogeneous, with variation in study designs, settings, professional groups and outcomes, limiting direct comparison across studies and precluding meta‐analysis. Methodological limitations were also identified in several included studies, particularly among cross‐sectional studies, which reduce confidence in some individual findings.

Eight of the 13 included studies were conducted during the COVID‐19 period, and several examined practices in relation to COVID‐19 specifically. These findings should therefore be interpreted within the exceptional clinical, resource, and workforce conditions of the pandemic and may not be fully transferable to routine perioperative practice or other transmissible infections. The geographical and clinical diversity of the included studies also means that organisational structures, resource availability, and IPC practices may differ across healthcare systems and operating room contexts. The small number of qualitative studies also limited in‐depth exploration of the contextual and experiential influences on IPC practice. These limitations highlight the need for further multidisciplinary research examining IPC practices for transmissible infections across different operating room settings and team members.

## 7 Conclusion

This review systematically searched for and synthesised findings from studies which investigated the influences of IPC practices for transmissible infections among operating room team members. An analysis of the findings revealed that IPC practices are mainly influenced by organisational, professional and educational factors along with practical challenges in implementing precautions. However, the heterogeneity and predominantly observational nature of the evidence limit certainty regarding the relative importance of these influences and the effectiveness of specific strategies to address them. These findings contribute to understanding the factors that may support or hinder perioperative infection prevention practice and highlight the role of perioperative nurses in communicating infection risks, implementing precautions and coordinating infection prevention activities across the multidisciplinary team. They also emphasise the importance of nursing education and leadership in supporting context‐appropriate IPC practice through education, appropriate resources and organisational support. Addressing these interconnected influences may help strengthen the translation of IPC recommendations into everyday operating room practice and support the safety of patients and healthcare workers.

## Author Contributions

**Kavitha Sriharan:** conceptualization, investigation, writing – original draft, methodology, validation, visualization, writing – review and editing, software, formal analysis, project administration, data curation, resources. **Rona Pillay:** conceptualization, investigation, methodology, validation, visualization, writing – review and editing, software, formal analysis, project administration, supervision, data curation, resources. **Paul Glew:** conceptualization, investigation, methodology, validation, visualization, writing – review and editing, software, formal analysis, project administration, data curation, supervision, resources. **Gift Mutsonziwa:** conceptualization, investigation, methodology, validation, visualization, writing – review and editing, software, formal analysis, project administration, data curation, supervision, resources.

## Funding

The authors have nothing to report.

## Ethics Statement

The authors have nothing to report.

## Conflicts of Interest

The authors declare no conflicts of interest.

## Supporting information


**Appendix S1:** Search terms and combinations.

**Appendix S2:** Studies that appeared to meet the inclusion criteria but were excluded, with reasons for exclusion.

**Appendix S3:** Summary of quality assessment.

**Appendix S4:** Development of categories and themes.

## Data Availability

The data that supports the findings of this study are available in Supporting Information of this article.
