# Supplementary material for: Influences of Infection Prevention and Control Practices for Transmissible Infections Among Operating Room Team Members: A Systematic Review
Source: Nurs Open. 2026 Oct 22;13(10):e70956. doi: 10.1002/nop2.70956 (PMC13690298; doi:10.1002/nop2.70956)
Supplement: Supplementary file 1 [file NOP2-13-e70956-s002.docx]

**Supplementary Appendix 3: Summary of quality assessment**

**Table 4.1: Critical Appraisal using JBI Checklist for Qualitative Studies (Lockwood et al., 2015)**

| **Author (year)** | **Q1** | **Q2** | **Q3** | **Q4** | **Q5** | **Q6** | **Q7** | **Q8** | **Q9** | **Q10** | **Total of ‘Yes’ in an article (%)** |
| --- | --- | --- | --- | --- | --- | --- | --- | --- | --- | --- | --- |
| Elliott et al. (2021) | Y | Y | Y | Y | Y | Y | Y | Y | Y | Y | 100 |
| Sürme et al. (2022) | U | Y | Y | Y | Y | N | N | Y | Y | Y | 70 |

Note:

Y: yes; U: unclear; N: no; N/A: not applicable

Q1: Is there congruity between the stated philosophical perspective and the research methodology?

Q2: Is there congruity between the research methodology and the research question or objectives?

Q3: Is there congruity between the research methodology and the methods used to collect data?

Q4: Is there congruity between the research methodology and the representation and analysis of data?

Q5: Is there congruity between the research methodology and the interpretation of results?

Q6: Is there a statement locating the researcher culturally or theoretically?

Q7: Is the influence of the researcher on the research, and vice-versa, addressed?

Q8: Are participants, and their voices, adequately represented?

Q9: Is the research ethical according to current criteria or, for recent studies, is there evidence of ethical approval by an appropriate body?

Q10: Do the conclusions drawn in the research report flow from the analysis, or interpretation, of the data?

**Table 4.2: Critical Appraisal using JBI Critical Appraisal Checklist for Analytical Cross-Sectional Studies (Barker et al., 2026)**

| **Author (year)** | **Q1** | **Q2** | **Q3** | **Q4** | **Q5** | **Q6** | **Q7** | **Q8** | **Total of ‘Yes’ in an article (%)** |
| --- | --- | --- | --- | --- | --- | --- | --- | --- | --- |
| Belgacem et al. (2023) | Y | N/A | Y | U | N | N | Y | Y | 57.1 |
| Eser & Aygün  (2024) | Y | Y | U | U | N | N | Y | Y | 50 |
| Lotfi et al. (2021) | Y | N/A | U | U | N | N | Y | Y | 42.9 |
| Prakash et al. (2020) | U | N/A | U | Y | N | N | U | Y | 28.6 |
| Ünal et al. (2025) | U | Y | N/A | Y | N/A | N/A | Y | Y | 80 |

Note:

Y: yes; U: unclear; N: no; N/A: not applicable

Q1: Were the criteria for inclusion in the sample clearly defined?

Q2: Were objective, standard criteria used for measurement of the condition?

Q3: Was the exposure measured in a valid and reliable way?

Q4: Were outcomes measured in a valid and reliable way?

Q5: Were confounding factors identified?

Q6: Were strategies to deal with confounding factors stated?

Q7: Was appropriate statistical analysis used?

Q8: Were the study subjects and the setting described in detail?

**Table 4.3: Critical Appraisal using JBI Critical Appraisal Checklist for Cohort Studies (Moola et al., 2020)**

| **Author (year)** | **Q1** | **Q2** | **Q3** | **Q4** | **Q5** | **Q6** | **Q7** | **Q8** | **Q9** | **Q10** | **Q11** | **Total of ‘Yes’ in an article (%)** |
| --- | --- | --- | --- | --- | --- | --- | --- | --- | --- | --- | --- | --- |
| Wall et al. (2022) | Y | Y | Y | Y | Y | Y | Y | Y | Y | N/A | Y | 100 |

Note:

Y: yes; U: unclear; N: no; N/A: not applicable

Q1: Were the two groups similar and recruited from the same population?

Q2: Were the exposures measured similarly to assign people to both exposed and unexposed groups?

Q3: Was the exposure measured in a valid and reliable way?

Q4: Were confounding factors identified?

Q5: Were the strategies to deal with confounding factors stated?

Q6: Were the groups/participants free of the outcome at the start (or at the moment of exposure)?

Q7: Were the outcomes measured in a valid and reliable way?

Q8: Was the follow up time reported and sufficient to be long enough for outcomes to occur?

Q9: Was follow up complete, and if not, were the reasons to loss to follow up described and explored?

Q10: Were the strategies to address incomplete follow up utilised?

Q11: Was appropriate statistical analysis used?

**Table 4.4: Critical Appraisal using the JBI Critical Appraisal Checklist for Quasi-Experimental Studies (Barker et al., 2024)**

| **Author (year)** | **Q1** | **Q2** | **Q3** | **Q4** | **Q5** | **Q6** | **Q7** | **Q8** | **Q9** | **Total of ‘Yes’ in an article (%)** |
| --- | --- | --- | --- | --- | --- | --- | --- | --- | --- | --- |
| Chia et al. (2024) | Y | N | U | U | Y | N/A | Y | Y | Y | 62.5 |
| Lacourciere et al. (2019) | Y | N/A | N/A | N/A | N | U | U | U | N/A | 20 |
| Munoz-Price et al. (2012) | Y | N/A | Y | Y | Y | Y | Y | Y | Y | 100 |

Note:

Y: yes; U: unclear; N: no; N/A: not applicable

Q1: Is it clear in the study what is the “cause” and what is the “effect”?

Q2: Was there a control group?

Q3: Were participants included in any comparisons similar?

Q4: Were the participants included in any comparisons receiving similar treatment/care, other than the exposure or intervention of interest?

Q5: Were there multiple measurements of the outcome, both pre and post the intervention/exposure?

Q6: Were the outcomes of participants included in any comparisons measured in the same way?

Q7: Were outcomes measured in a reliable way?

Q8: Was follow-up complete and if not, were differences between groups in terms of their follow-up adequately described and analysed?

Q9: Was appropriate statistical analysis used?

**Table 4.5: Critical Appraisal using the JBI Critical Appraisal Checklist for Randomised Controlled Trials (Barker et al., 2023)**

| **Author (year)** | **Q1** | **Q2** | **Q3** | **Q4** | **Q5** | **Q6** | **Q7** | **Q8** | **Q9** | **Q10** | **Q11** | **Q12** | **Q13** | **Total of ‘yes’ in an article (%)** |
| --- | --- | --- | --- | --- | --- | --- | --- | --- | --- | --- | --- | --- | --- | --- |
| Stutz et al. (2023) | Y | U | Y | N | U | U | N | N | Y | Y | Y | Y | Y | 53.8% |

Note:

Y: yes; U: unclear; N: no; N/A: not applicable

Q1: Was true randomisation used for the assignment of participants to treatment groups?

Q2: Was allocation to treatment groups concealed?

Q3: Were treatment groups similar at the baseline?

Q4: Were participants blind to the treatment assignment?

Q5: Were those delivering treatment blind to treatment assignment?

Q6: Were outcomes assessors blind to treatment assignment?

Q7: Were treatment groups treated identically other than the intervention of interest?

Q8: Was follow up complete and if not, were differences between groups in terms of their follow up adequately described and analysed?

Q9: Were participants analysed in the groups to which they were randomised?

Q10: Were outcomes measured in the same way for treatment groups?

Q11: Were outcomes measured in a reliable way?

Q12: Was appropriate statistical analysis used?

Q13: Was the trial design appropriate, and any deviations from the standard RCT design (individual randomisation, parallel groups) accounted for in the conduct and analysis of the trial?

**Table 4.6: Critical Appraisal using the Mixed Methods Appraisal Tool (MMAT) (Hong et al., 2018)**

| **Category of study designs** | **Methodological quality criteria** |  |
| --- | --- | --- |
|  |  | **Cutter & Jordan (2012)** |
| Screening (for all types) | S1 Are there clear research questions? | Y |
|  | S2 Do the collected data allow to address the research question? | Y |
| 1. Qualitative component | 1.1 Is the qualitative approach appropriate to answer the research question? | Y |
|  | 1.2 Are the qualitative data collection methods adequate to address the research question? | Y |
|  | 1.3 Are the findings adequately derived from the data? | Y |
|  | 1.4 Is the interpretation of results sufficiently substantiated by data? | Y |
|  | 1.5 Is there coherence between qualitative data sources, collection, analysis and interpretation? | Y |
| 3. Quantitative non-randomised component | 3.1 Are the participants representative of the target population? | Y |
|  | 3.2 Are the measurements appropriate regarding both the outcome and intervention (or exposure)? | Y |
|  | 3.3 Are there complete outcome data? | Y |
|  | 3.4 Are the confounders accounted for in the design and analysis? | Y |
|  | 3.5 During the study period, is the intervention administered (or exposure occurred) as intended? | Y |
| 4. Quantitative descriptive component | 4.1 Is the sampling strategy relevant to address the research question? | - |
|  | 4.2 Is the sample representative of the target population? | - |
|  | 4.3 Are the measurements appropriate? | - |
|  | 4.4 Is the risk of nonresponse bias low? | - |
|  | 4.5 Is the statistical analysis appropriate to answer the research question? | - |
| 5. Mixed methods component | 5.1 Is there an adequate rationale for using a mixed methods design to address the research question? | Y |
|  | 5.2 Are the different components of the study effectively integrated to answer the research question? | Y |
|  | 5.3 Are the outputs of the integration of qualitative and quantitative components adequately interpreted? | Y |
|  | 5.4 Are divergences and inconsistencies between quantitative and qualitative results adequately addressed? | Y |
|  | 5.5 Do the different components of the study adhere to the quality criteria of each tradition of the methods? | Y |

Note:

Y: yes; C: can’t tell; N: no
