# Supplementary material for: Influences of Infection Prevention and Control Practices for Transmissible Infections Among Operating Room Team Members: A Systematic Review
Source: Nurs Open. 2026 Oct 22;13(10):e70956. doi: 10.1002/nop2.70956 (PMC13690298; doi:10.1002/nop2.70956)
Supplement: Supplementary file 2 [file NOP2-13-e70956-s004.docx]

**Supplementary Appendix 1: Search Terms and Combinations**

**1. CINAHL Ultimate (EBSCOhost) (15 August 2026)**

| #1 | ["infection control" OR "infection prevention" OR "cross infection" OR "infection transmission" OR "transmission based precaution*" OR "transmission-based precaution*" OR isolation OR precaution*](https://research.ebsco.com/c/rhbbo2/search/advanced-results?db=cul&expanders=concept&limiters=None&q=%22infection%20control%22%20OR%20%22infection%20prevention%22%20OR%20%22cross%20infection%22%20OR%0A%22infection%20transmission%22%20OR%20%22transmission%20based%20precaution%2A%22%20OR%0A%22transmission-based%20precaution%2A%22%20OR%20isolation%20OR%20precaution%2A&searchMode=all&searchQueryId=sq%3A19756c4b-427d-44a3-a30f-1412b4359eb4&sort=relevance&userDirectAction=true)” |
| --- | --- |
| #2 | ["operating room*" OR "operating theatre*" OR "operating department*" OR "perioperative team*" OR "perioperative staff" OR "perioperative personnel"](https://research.ebsco.com/c/rhbbo2/search/advanced-results?db=cul&expanders=concept&limiters=None&q=%22operating%20room%2A%22%20OR%20%22operating%20theatre%2A%22%20OR%20%22operating%20department%2A%22%20OR%0A%22perioperative%20team%2A%22%20OR%20%22perioperative%20staff%22%20OR%20%22perioperative%20personnel%22&searchMode=all&searchQueryId=sq%3Ae4f792b5-c200-49b1-87c5-f28b838e3881&sort=relevance&userDirectAction=true) |
| #3 | "operating room personnel" OR "operating room staff" OR "operating theatre staff" OR "surgical team*" OR "perioperative team*" OR "perioperative personnel" OR "perioperative staff" OR "operating room nurs*" OR "operating theatre nurs*" OR surgeon* OR anesthe* OR anaesthe* |
| #4 | #1 AND #2 AND #3 FILTERS : English, from 2010-2026 |
| Results | 510 |

**2. APA PsycInfo (Ovid) (15 August 2026)**

| #1 | ("infection control" or "infection prevention" or "cross infection" or "infection transmission" or "transmission based precaution*" or "transmission-based precaution*" or isolation or precaution*).mp. [mp=title, abstract, heading word, table of contents, key concepts, original title, tests & measures, mesh word] |
| --- | --- |
| #2 | ("operating room*" or "operating theatre*" or "operating department*" or perioperative).mp. [mp=title, abstract, heading word, table of contents, key concepts, original title, tests & measures, mesh word] |
| #3 | ("operating room personnel" or "operating room staff" or "operating theatre staff" or "surgical team*" or "perioperative team*" or "perioperative personnel" or "perioperative staff" or "operating room nurs*" or "operating theatre nurs*" or "health care worker*" or "healthcare worker*" or nurs* or surgeon* or physician* or doctor* or anesthe* or anaesthe*).mp. [mp=title, abstract, heading word, table of contents, key concepts, original title, tests & measures, mesh word] |
| #4 | #1 AND #2 AND #3 LIMIT: English language, yr=”2010-Current” |
| Results | 17 |

**3. Scopus (15 August 2026)**

|  | TITLE-ABS-KEY ( "infection control" OR "infection prevention" OR "cross infection" OR "infection transmission" OR "transmission based precaution*" OR "transmission-based precaution*" OR isolation OR precaution* ) AND TITLE-ABS-KEY ( "operating room*" OR "operating theatre*" OR "operating department*" OR "perioperative team*" OR "perioperative staff" OR "perioperative personnel" ) AND TITLE-ABS-KEY ( "operating room personnel" OR "operating room staff" OR "operating theatre staff" OR "surgical team*" OR "perioperative team*" OR "perioperative personnel" OR "perioperative staff" OR "operating room nurs*" OR "operating theatre nurs*" OR surgeon* OR anesthe* OR anaesthe* ) AND ( LIMIT-TO ( LANGUAGE , "English" ) ) AND PUBYEAR > 2009 AND PUBYEAR < 2027 |
| --- | --- |
| Results | 977 |

**4. PubMed (15 August 2026)**

| #1 | "Infection Control"[MeSH Terms] OR "Cross Infection"[MeSH Terms] OR "infection control"[Title/Abstract] OR "infection prevention"[Title/Abstract] OR "cross infection"[Title/Abstract] OR transmission[Title/Abstract] OR "transmission based precaution*"[Title/Abstract] OR "transmission-based precaution*"[Title/Abstract] OR isolation[Title/Abstract] OR precaution*[Title/Abstract] |
| --- | --- |
| #2 | "Operating Rooms"[MeSH Terms] OR "operating room*"[Title/Abstract] OR "operating theatre*"[Title/Abstract] OR "operating department*"[Title/Abstract] OR "perioperative team*"[Title/Abstract] OR "perioperative staff"[Title/Abstract] OR "perioperative personnel"[Title/Abstract] |
| #3 | "Health Personnel"[MeSH Terms] OR "operating room personnel"[Title/Abstract] OR "operating room staff"[Title/Abstract] OR "operating theatre staff"[Title/Abstract] OR "surgical team*"[Title/Abstract] OR "perioperative team*"[Title/Abstract] OR "perioperative staff"[Title/Abstract] OR "perioperative personnel"[Title/Abstract] OR "operating room nurs*"[Title/Abstract] OR "operating theatre nurs*"[Title/Abstract] OR surgeon*[Title/Abstract] OR anesthe*[Title/Abstract] OR anaesthe*[Title/Abstract] |
| #4 | #1 AND #2 AND #3 Filters: English, from 2010 - 2026 |
| Results | 803 |

**5. Web of Science (15 August 2026)**

| #1 | "infection control" OR "infection prevention" OR "cross infection" OR "infection transmission" OR "transmission based precaution*" OR "transmission-based precaution*" OR isolation OR precaution* (Topic) |
| --- | --- |
| #2 | ("operating room*" OR "operating theatre*" OR "operating department*" OR "perioperative team*" OR "perioperative staff" OR "perioperative personnel") (Topic) |
| #3 | ("operating room personnel" OR "operating room staff" OR "operating theatre staff" OR "surgical team*" OR "perioperative team*" OR "perioperative personnel" OR "perioperative staff" OR "operating room nurs*" OR "operating theatre nurs*" OR surgeon* OR anesthe* OR anaesthe*) (Topic) |
| #4 | #1 AND #2 AND #3 Limits: English, from 2010-2026 |
| Results | 458 |
